# Diet, occupational exposure and early asthma incidence among bakers, pastry makers and hairdressers

**DOI:** 10.1186/1471-2458-12-387

**Published:** 2012-05-29

**Authors:** Thomas Rémen, Dovi-Stéphanie Acouetey, Christophe Paris, Denis Zmirou-Navier

**Affiliations:** 1 Inserm U954 (Institut National de la santé et de la Recherche Médicale), School of Medicine, Nancy, France; 2 Lorraine University Medical School, Nancy, France; 3 EHESP School of Public Health, Sorbonne-Paris Cité, Rennes, France; 4 INSERM U954, Faculté de Médecine, Bâtiment E, 2ème étage, 9 avenue de la forêt de Haye, 54505, VANDOEUVRE-LES-NANCY, France

**Keywords:** Occupational asthma, Epidemiology, Atopy, Vitamins

## Abstract

**Background:**

The natural history of occupational asthma (OA) is influenced by many determinants. This study aims to assess the combined roles of personal characteristics, including occupational exposure and nutritional habits, on the incidence of OA during the first years at work.

**Methods:**

A nested case–control study was conducted within a retrospective cohort of young workers in the bakery, pastry-making and hairdressing sectors. Cases were subjects diagnosed as ‘confirmed’ or ‘probable’ OA consecutively to a medical visit (N = 31). Controls were subjects without OA (N = 196). Atopy was defined after blood specific IgE analysis, based on the Phadiatop^TM^ test. Occupational exposure was characterized by standardized questionnaires and diet patterns by a food frequency questionnaire.

**Results:**

Among bakers and pastry-makers, only atopy is an independent risk factor of OA (OR = 10.07 95%CI [2.76 – 36.65]). Among hairdressers, several variables are associated with OA. Body mass index (unit OR = 1.24 [1.03 – 1.48]) and the score of exposure intensity (unit OR = 1.79 [1.05 – 3.05]) are independent predictors of OA, but the role of atopy is weak (OR = 4.94 [0.66 – 36.75]). Intake of vitamin A is higher among hairdressers cases (crude p = 0.002, adjusted p = 0.01 after control for body mass index and atopy); the same observation is made for vitamin D (crude p = 0.004, adjusted p = 0.01).

**Conclusion:**

This study suggests that the influence of several factors on the incidence of OA, including dietary vitamins, might vary across exposure settings.

## Background

Occupational asthma (OA) is a disease characterized by variable airflow limitation and/or airway hyperresponsiveness due to causes and conditions attributable to a particular work environment rather than to stimuli encountered outside the workplace [1]. Two types of OA can be distinguished: (i) immunologic OA appears after a latency period of exposure necessary for acquiring immunologic sensitization to the causal agent(s); and (ii) non immunologic OA occurs after acute exposure to high concentrations of irritants (‘irritant-induced asthma’) [2].

A general framework for the natural history of immunological OA encompasses several stages after onset of exposure, including development of sensitization and inception of OA that can be followed by removal from exposure and remission or persistence of OA [3]. This allergic march is influenced by multiple determinants. While occupational exposure plays the key role, with the interplay between the nature of agents encountered at the workplace (in particular the contrast between high– vs. low molecular weight agents) and intensity or exposure duration before and after occurrence of symptoms, other factors related to personal or more general characters also contribute to the onset of the disease, among which are genetic predispositions and possibly nutritional factors [3].

A large increase in the prevalence of asthma was observed during the last decades in most developed countries [4]. According to Allan *and coll.*, it would be the consequence of changing environmental and/or lifestyle factors rather than genetic influences [5]. Changes in diet have been put forward as responsible for part of this increase. However, interactions between these factors remain unclear. Numerous hypotheses have been discussed concerning the role of nutrition in asthma occurrence without definitive evidence. The role of antioxidants (vitamins A, C, E) or of vitamin D is still questioned, with contradictory hypotheses being reported [5,6]. Considering poly-unsaturated fatty acids (PUFA), a suggested mechanism relates increased dietary n-6:n-3 PUFA ratio to the augmentation of allergic disease and to asthma [4]. These hypotheses still need to be documented. Most of the epidemiologic studies involving nutritional factors have been implemented in the general population. Very few explored nutrition-environment interactions in OA [7] which can be viewed as a model of “experimental” asthma [8].

In the framework of a retrospective follow-up of young bakers, pastry-makers and hairdressers, i.e. in occupational sectors with a known high risk of OA, a nested case–control study was undertaken to assess the combined influence of personal characteristics (including nutritional habits) and occupational exposure on the incidence of OA during the first years at work.

## Methods

### Design and study population

The study protocol has been published previously [9]. Briefly, the ABCD (French acronym for early asthma in bakery and hairdressing sectors) study aimed to assess early incidence of OA among young workers according to their sector of activity and exposure duration, and to identify risk factors of OA. It was based on a retrospective follow-up design, with a nested case–control facet. The population based study comprises groups of exposed (bakers, pastry-makers and hairdressers) and non exposed subjects (sales and food sectors: butcher, pork butcher, caterer, cook job…), used as a reference group, who graduated between 2001 and 2006 (2001 for the non exposed group) from nine vocational schools in Lorraine, North-Eastern France. The cohort consisted of all who had completed a phone medical questionnaire about respiratory, ENT and skin conditions since engaging in their training and occupation, and about their connection with work [10].

Within this subset of the cohort, all subjects who declared work-related respiratory symptoms or isolated work-related rhinitis symptoms that had appeared after inception of exposure, and a matched sample of all others (frequency matching criteria: year of graduation, vocational school and occupational sector) were invited to participate in a medical visit to complete clinical and lung function investigations and to collect blood samples for IgE assays (total IgE and specific IgE for work-related and common allergens).

The research program is authorized by the Nancy University Hospital ethics committee and written consents are obtained from the young workers themselves.

### Selection of cases and controls

For the nested case–control study, we included only bakers, pastry-makers and hairdressers who had the medical visit.

Diagnosis of OA encompassed two aspects: (i) diagnosis of asthma, and (ii) evidence of a temporal relationship between the occurrence of symptoms and occupation. Each subject who performed the medical visit was classified according to a decisional tree for the definition of OA [10] into one of four categories: (i) *confirmed OA*; (ii) *probable OA*; (iii) *possible OA;* and (iv) *absence of OA*. Briefly, this classification was established after data collected during the medical visit on the basis of the following criteria: (i) the clinical definition of asthma proposed by the International Primary Care Respiratory Group [11]; (ii) recording of peak-flow expiratory curves for three weeks; (iii) a spirometry with bronchodilatator testing (short acting β-2 agonist); and finally (iv) work-related specific IgE assays when available.

*Cases were subjects diagnosed as ‘confirmed’ or ‘probable’ OA.* Controls were subjects not diagnosed as OA *(neither confirmed, probable, nor possible)* and nor labelled with *‘unknown’* OA status (when PEF monitoring was not exploitable and reversibility tests were either not feasible or not usable).

### Data collection

During the medical examination, height and weight were recorded with the same device - electronic balance and electronic height gauge - for all subjects (to avoid measurement bias). Two investigators realized the vast majority of medical visits (TR and DSA), with the assistance of three medical interns for some visits. Data on past and present tobacco smoking, history of work-related respiratory, rhinitis, eczema and urticaria symptoms were collected using the EGEA questionnaire [12]. The time sequence of symptoms with work was assessed using items derived from other dedicated questionnaires [13,14]. Atopic family history (first degree) was considered if at least one of the following pathologies was reported: asthma, hay fever, eczema, urticaria, or in case of a family history of treatment for allergy or desensitisation.

From blood sampling, specific IgE analysis was used to categorize subjects as atopic or not, based on the Phadiatop^TM^ test (Phadia, Sweden). This test screens an IgE-dependant allergy with a median sensitivity of 96% (ranging from 70% to 100% across studies) and a median specificity of 95% (77% to 100%) according to a meta-analysis on a general adult population [15]. The Phadiatop^TM^ test includes mite, pollen, mould, and animal dander allergens, but the exact allergen composition is not issued by the company [15].

The phone screening questionnaire and the medical visit took place from March 2009 to July 2010. Analyses were performed from November 2010 to August 2011. The research program was authorized by the Nancy University Hospital ethics committee and written consents were obtained from the young workers themselves.

### Exposure assessment

Exposure scores and duration were obtained from the occupational exposure questionnaires [16,17]. A score of *exposure intensity* depends on the average number of tasks declared to be realized each day. For bakers, the daily tasks were bread-making (kneading-machine loading, transfer by shovel, dough division, dough shaping, bread put in the oven), pastry-making (pastry preparation) and cleaning activities, and were scored (0, 1 or 2) using tertiles [see Additional file 1: Table S1 and Table S2 for more precision]. The sum of these 7 notes (from 0 to 2) defined the score of exposure intensity, a score of 0 reflecting a low exposure, a score of 14 a very high exposure. Calculation of exposure duration entailed reconstruction of the occupational history of each subject since inception of apprenticeship (duration of apprenticeship, diplomas obtained, hiring date, cumulative length of unemployment, date of sector dropout and date of administration of the phone questionnaire). This exposure duration corresponds to the cumulative periods of exposure since engaging in apprenticeship (excluding classroom periods during apprenticeship or periods of inactivity) until the date of the telephone interview. Hence, by construction, one year exposure duration encompasses more than one calendar year.

For hairdressers, a corresponding intensity score was based on the following four activities: perm, hair dyes, hair bleached and rinsing, with a note of 0, 1 or 2 according to tertiles of the average number of hairdressing tasks reported per day. The sum of these 4 scores (from 0 to 2) defined the score of exposure intensity among hairdressers, a score of 0 reflecting a low exposure, a score of 8 a very high exposure [see Additional file 1: Table S1 and S2 for more precision].

### Nutritional intake

A food frequency questionnaire (Suvimax 2) [18] was used to evaluate dietary imbalance of nutritional factors during the 12 months prior to the medical visit. Briefly, this semi-quantitative food frequency questionnaire (FFQ) was developed for self-administered assessment of usual dietary intake over the past year among French adults at an individual level. The food list contained 240 food and beverage items categorized into 22 categories. Validity and reproducibility of this questionnaire has been described elsewhere [18]. The frequency of consumption referred to usual consumption over the past year on an increasing scale including yearly, monthly, weekly or daily units, as suitable. The FFQ was self-administered and completed at home. Questionnaires were recovered and verified during the medical visit in order to complete eventual missing information and correct ambiguities.

French recipes, validated by food and nutrition professionals, were used to estimate amounts of simple items consumed among mixed foods. Frequencies were converted into numbers of servings per day and multiplied by the standard portion size or by the portion size declared using photographs. An ad hoc composition table was developed to calculate weighted mean nutrient values. The weights were derived from the gender-specific mean frequency of each item that was declared [18].

### Statistical analysis

The data were analysed using SAS® 9.2 software. Proportions are expressed as percentages and quantitative data as means with standard deviations (SD). Quantitative data were tested for linearity. For non linear variables, they were divided into categories using quartiles. Comparisons used chi-square or Fisher exact tests, Student or Kruskal-Wallis tests, as appropriate. Logistic regression was used to calculate odds ratios (ORs) and 95% confidence intervals, while adjusting for confounders or exploring effect modification. The significance level to remain in the model was fixed to 0.20, not to overlook confounding and weak associations.

Personal characteristics such as gender, age, BMI and smoking habits were compared between cases and controls. Medical characteristics such as atopy, respiratory, ENT and skin symptoms were also compared between these two groups. The role of nutritional intakes was explored in a second step. Crude ORs and, when appropriate, ORs adjusted for potential confounders such as atopy and body mass index are presented. Work-related symptoms were not retained for the multivariate model because of their strong association with the OA status.

Because of differences in the mechanisms involved in the onset of OA between the two occupational sectors, determinants of OA can vary according to the sector. Hence, the analysis was conducted separately according to sector, after a global assessment.

## Results

### Demographics

31 cases met the inclusion criteria. Respiratory symptoms had begun between less than 1 year to 7.4 years prior to the medical visits. Among the 202 control subjects meeting the inclusion criteria, 6 were excluded because of airway responsiveness whose association with occupational nature could not be investigated (absence of specific IgE assays), bringing the total number to 196 controls. Demographic and other relevant characteristics of the 227 subjects (mean age: 25.2 years, 40.5% hairdressers) retained in the analysis are summarised in Table 1.

**Table 1:** Descriptive characteristics of cases and controls

**Characteristic**		**Cases (N = 31)**	**Controls (N = 196)**	**Total (N = 227)**	**p**	**Test used**^Ω^
Women	%	8 (25.8%)	94 (48.0%)	102 (44.9%)	**0.02**	Chi2
Age (years)	Mean [SD]	26.1 [2.5]	25.1 [3.0]	25.2 [2.9]	0.06	T
Height (cm)	Mean [SD]	173.4 [8.0]	170.1 [9.2]	170.5 [9.1]	0.06	T
Weight (kg)	Mean [SD]	77.5 [15.5]	70.2 [14.1]	71.2 [14.5]	**0.009**	T
Tobacco smoking (at visit):						
Never smokers	%	10 (32.2%)	76 (38.8%)	86 (37.9%)		
Ex smokers	%	7 (22.6%)	26 (13.3%)	33 (14.5%)	0.38	Chi2
Current smokers	%	14 (45.2%)	94 (48.0%)	108 (47.6%)		
Occupation:						
Hairdressers	%	7 (22.6%)	85 (43.4%)	92 (40.5%)	**0.03**	Chi2
Exposure duration (years)	Mean [SD]	6.1 [3.1]	6.7 [2.2]	6.6 [2.4]	0.25	KW
Sector dropout^†^	%	10 (32.3%)	22 (11.2%)	32 (14.1%)	**0.004**	F

Ω Chi2 = chi-square test – T = Student test – KW = Kruskal-Wallis test – Fisher = fisher test.

† From the sector in which the subjects graduated.

Differences were observed between cases and controls concerning occupational sector and gender (more hairdressers and therefore more women among controls, respectively p = 0.03 and p = 0.02) and sector dropout (higher among cases [p = 0.002]). Among hairdressers retained for the case–control study, 96.7% are women while, among bakers and pastry-makers, 90.4% are men. Because gender and occupational sectors are so strongly associated, no adjustment for sex will be done in sector specific analyses.

### Atopy, exposure and symptoms

Crude associations are summarised in Table 2, separately for bakers and pastry-makers on the one hand, and hairdressers on the other hand. Atopy is significantly more frequent among cases than among controls in the bakery and pastry-making sectors; the same pattern is seen in hairdressers, but fails to reach significance. Exposure intensity is higher among hairdressing cases.

**Table 2:** Distribution of symptoms and of putative risk factors among cases and controls according to the occupational sector

**Variable**		**All**			**Bakers and pastry-makers**			**Hairdressers**			
		**Cases (N = 31)**	**Controls (N = 196)**	**p**	**Cases (N = 24)**	**Controls (N = 111)**	**p**	**Cases (N = 7)**	**Controls (N = 85)**	**p**	**Test used**^**Ω**^
Body mass index (kg/m^2^)	Mean [SD]	25.9 [5.9]	24.2 [3.9]	0.13	25.6 [5.8]	25.1 [4.1]	0.94	27.2 [6.3]	23.1 [3.4]	0.07	KW
Work-related rhinitis symptoms	%	28 (90.3%)	77 (39.3%)	**<0.001**	22 (91.7%)	45 (40.5%)	**<0.001**	6 (85.7%)	32 (37.7%)	**0.02**	Chi2
Work-related eczema symptoms	%	11 (35.5%)	23 (11.7%)	**0.002**	7 (29.2%)	7 (6.4%)	**<0.001**	4 (57.1%)	16 (18.8%)	**0.04**	Chi2
Work-related urticaria symptoms	%	7 (22.6%)	12 (6.1%)	**0.007**	6 (25.0%)	4 (3.6%)	**0.002**	1 (14.3%)	8 (9.4%)	0.53	Fisher
Personal atopy	%	26 (83.9%)	78 (39.8%)	**<0.001**	21 (87.5%)	44 (39.6%)	**<0.001**	5 (71.4%)	34 (40.0%)	0.13	Chi2
Family atopy	%	12 (38.7%)	82 (41.8%)	0.74	8 (33.3%)	37 (33.3%)	1.00	4 (57.1%)	45 (52.9%)	1.00	Chi2
Exposure duration (year)											
0 - 4.7 (Q1)	%	14 (45.1%)	42 (21.4%)		11 (45.8%)	29 (26.1%)		3 (42.9%)	13 (15.3%)		
4.8 - 6.6 (Q2)	%	3 (9.7%)	55 (28.1%)	**0.02**	2 (8.3%)	33 (29.7%)	0.11	1 (14.3%)	22 (25.9%)	0.40	Chi2
6.7 - 8.2 (Q3)	%	7 (22.6%)	49 (25.0%)		6 (25.0%)	25 (22.5%)		1 (14.3%)	24 (28.2%)		
8.3 - 13.8 (Q4)	%	7 (22.6%)	50 (25.5%)		5 (20.8%)	24 (21.6%)		2 (28.6%)	26 (30.6%)		
Score of exposure intensity											
Among bakers/pastrymakers§	Mean [SD]	-	-	-	5.0 [3.1]	5.5 [3.5]	0.59	-	-	-	KW
Among hairdressers†	Mean [SD]	-	-	-	-	-	-	6.9 [1.3]	4.5 [2.4]	**0.01**	KW

Ω KW = Kruskal-Wallis test – Chi2 = chi-square test – Fisher = fisher test.

§ Score max = 14.

† Score max = 8.

After multivariate analysis, only atopy remains an independent risk factor of OA among bakers and pastry-makers (OR = 10.07 95%CI [2.76 – 36.65]). Among hairdressers, atopy is weakly associated with OA (OR = 4.94 [0.66 – 36.75]), while BMI (unit OR = 1.24 [1.03 – 1.48]) and the score of exposure intensity (unit OR = 1.79 [1.05 – 3.05]) are independent predictors of OA.

### OA and nutrient intakes

Associations between OA and nutrients intake are presented in Table 3. Overall, the associations of OA with vitamins D and E are borderline significant (p = 0.06 for the two vitamins: 3.8 μg [SD = 3.0] among cases, vs 2.9 μg [2.1] among controls for vitamin D; respectively 21.1 mg [16.7] and 16.3 mg [11.0] for vitamin E), but vanishes after adjustment for body mass index and personal atopy. Adding exposure intensity in the model does not change the effect measures of nutrients intake (data not shown).

**Table 3:** Associations between OA and nutrients intake, by occupational sector

**Nutrient intake**	**Unit**	**All**		**Bakers and pastry makers**		**Hairdressers**	
		Crude odds ratio*	**Adjusted**^**†**^odds ratio*	Crude odds ratio*	**Adjusted**^**†**^odds ratio*	Crude odds ratio*	**Adjusted**^**†**^odds ratio*
Energy intake	Mcal	**1.33 [1.07 - 1.65]**	**1.28 [1.01 - 1.60]**	1.12 [0.86 - 1.44]	1.07 [0.81 - 1.41]	**2.16 [1.15 - 4.07]**	**1.94 [1.00 - 3.72]**
n-6:n-3 PUFA ^λ^	g	1.02 [0.91 - 1.13]	1.01 [0.91 - 1.13]	1.11 [0.96 - 1.28]	1.08 [0.92 - 1.25]	0.77 [0.56 – 1.06]	0.74 [0.50 - 1.10]
Vitamin A	mg	1.16 [0.89 - 1,52]	1.22 [0.90 - 1.65]	0.93 [0.61 - 1.41]	0.90 [0.50 - 1.60]	**4.08 [1.64 - 10.14]**	**3.47 [1.30 - 9.22]**
Vitamin C	dg	1.04 [0.81 - 1.33]	1.00 [0.76 - 1.33]	1.04 [0.78 - 1.39]	1.00 [0.69 - 1.43]	1.05 [0.63 - 1.73]	1.08 [0.63 - 1.85]
Vitamin D	μg	1.15 [0.99 - 1.35]	1.13 [0.95 - 1.34]	1.01 [0.83 - 1.23]	1.01 [0.82 - 1.23]	**1.75 [1.20 - 2.56]**	**1.71 [1.12 - 2.59]**
Vitamin E	cg	1.30 [0.98 - 1.71]	1.23 [0.90 - 1.67]	1.20 [0.86 - 1.68]	1.09 [0.76 - 1.57]	1.49 [0.89 - 2.50]	1.64 [0.91 - 2.94]

^*^ OR per unit increase of each variable.

^†^ For personal atopy and body mass index.

^λ^ Ratio polyunsaturated fatty acids of omega 6 (n-6 PUFA)/polyunsaturated fatty acids of omega 3 (n-3 PUFA).

Among bakers and pastry-makers alone, no nutrient intake is associated with OA, whether prior to or after adjustment. On the other hand, among hairdressers, intake of vitamin A is higher among OA cases before adjustment (p = 0.002; 1.9 mg [1.2] among cases, vs 0.7 μg [0.6] among controls) and after (p = 0.01). The same association is found with vitamin D, whether before (p = 0.004; 5.5 μg [2.5] among cases, vs 2.5 μg [1.7] among controls) or after adjustment (p = 0.01).

## Discussion

Our results show that among hairdressers, intensity of exposure, measured by the daily number of specific tasks, and body mass index are risk factors of OA. Also, vitamin A and D intakes are greater among cases than among controls in this group. On the other hand, nutritional patterns showed no association with OA among bakers and pastry-makers. While the well known role of atopy on the occurrence of OA is confirmed in this sector, it is also suggested among hairdressers.

In our study, “work intensity” is positively associated with OA incidence among hairdressers and reflects the number and variety of techniques (discoloration, colouring, perms) performed in a typical day. Akpinar-Elci and coworkers also found a higher risk of OA in high work intensity hairdressers [19], in accord with another study that showed an increased risk of OA, thought not significant, among hairdressers often performing hair bleaching treatments or using hair spray, compared with more infrequent users [20]. We found no such association among bakers and pastry-makers. For Brisman *et al.*, the risk of asthma or rhinitis is less dependent on the cumulative dose of inhaled flour dust than on current exposure [21]; also, a longitudinal study showed significant associations between dust concentrations at onset of disease and the risk of asthma and rhinitis [22]. Discrepancies in the job dropout process due to OA symptoms could in part explain these differences in the time sequence between exposure and disease incidence across occupational sectors, since bakers have more opportunities for job reclassification than hairdressers, in particular by switching to pastry-making, an activity where exposure levels are lower [23], or to industrial bread processing rather than in the small, solo-bakeries that composed the vast majority of our study settings.

BMI was positively associated with OA among our study hairdressers (unit OR = 1.24 [1.03 – 1.48]). While data on OA are scarce, many studies explored the possible link between BMI and non occupational asthma or airway inflammation [24-31], showing a positive association where weight gain occurs before the onset of asthma and respiratory symptoms. This relationship between BMI and asthma could depend on gender, with a greater risk among women [32,33]. This might explain why we found an association between BMI and asthma in hairdressers, a predominantly female population, and not among bakers/pastry-makers, a predominantly male population.

Atopy is a well known risk factor of work-related sensitization, especially for high molecular weight agents. The odds ratio among bakers/pastry-makers is high (10.07 [2.76 - 36.65]). Among hairdressers, the association is more uncertain, possibly due to small numbers (OR = 4.94 [0.66 - 36.75]), but the role of atopy is still controversial in the literature [34].

In occupational settings, nutritional factors could significantly modify host responses to environmental toxicants. According to Romieu *and coll.*, “an adequate diet may inhibit, arrest, or even reverse the chain of events in toxicity, while a deficient diet could increase persons’ susceptibility to adverse environmental exposures, such as occupational allergens” [35].

To the best of our knowledge, this is the first study that explored the association between nutrient intakes, vitamins in particular, and OA. There are controversies about the relation between diet antioxidant intake and asthma [5]. Some authors assert that the increase of asthma is a consequence of a decline in antioxidant intake associated with the transition from a traditional to the modern diet [36]. An experimental study conducted by Gu found that supplementation with antioxidant vitamins in toluene diisocyanate-treated animals, mimicking an occupational exposure, ameliorated the respiratory eosinophilia [7]. In contrast, our results are consistent with the hypothesis that the increase in asthma and allergic diseases is favoured by an enhanced antioxidant intake associated with the greater availability of functional and antioxidant-enriched foods that might switch the balance of Th1 and Th2 towards a Th2 response [37].

Our findings suggest that the role of nutritional factors might depend on the exposure context. While exposure to flour dust and atopy, but not diet, are the key predictors of OA among bakers and pastry makers, nutritional factors are associated with OA among hairdressers, subjects who encounter several pro-oxidant chemicals agents in the work place. In a normal lung, there is a balance between the toxicity of oxidants (generated through normal cellular function or exposure to an oxygen-rich environment) and the protective activities of several intracellular and extracellular antioxidant defense systems [38]. Oxidative stress occurs when there is an imbalance between the antioxidant defense system of the body and oxidant insults, such as cigarette smoke, air pollution and infections [35]. Chemicals agents may cause disequilibrium in this balance, through an increase in oxidant stress and a compromise of antioxidant resources, and this might result in pathophysiologic events in the lung that culminate in cellular death and pulmonary dysfunction [35].

Concerning vitamin D, results are also contradictory in the literature: a first hypothesis proposed that the increase in allergy and asthma is a consequence of widespread vitamin D insufficiency [39]. A second one, more in line with our results, links this increase to a widespread early life vitamin D supplementation for rickets prophylaxis in developed countries [39]; now, high dose in vitro vitamin D supplementation was shown to promote Th2 differentiation [40]. Another study also showed that vitamin D is associated with a dose-dependent reduction in transcription of Th1 cytokines, and increased expression of the Th2 cytokines [41]. Studies on molecular epigenetic mechanisms of dietary vitamin D in lung cellular function (senescence, apoptosis, autophagia, proliferation, phagocytosis) are still ongoing. Such data might shed light on how dietary vitamin D supplementation might interact with environmental agents and give place to chronic lung diseases like asthma [42]. Prospective studies of supplementation therapy or sub-optimal nutrient intake are needed to confirm these hypotheses.

### Strengths and weaknesses of the study

The main limitation is the small number of cases which affects the statistical power of our study, possibly explaining the unstable (non significant) association between atopy and OA among hairdressers where absence of biomarkers of sensitization (specific IgE) precluded a clear assessment of asthma-like symptoms in relation to work. As in all case–control studies with other than incident cases, one should be cautious about the measures of associations since the information is collected some time after declaration of the disease. Although nested in a cohort, cases had started their symptoms with varying anteriority, due to the retrospective nature of the cohort. Thus, assessment of some risk factors might be affected by potential changes in behaviours or practices following onset of the disease (e.g. regarding the smoking status, the occupational tasks that are performed or dietary habits).

Strengths of this study lie in its design, with cases and controls coming from the same retrospective cohort study, in the accurate exposure duration history and in the diagnostic criteria we used for OA ascertainment. An interesting aspect of our study stems from the comparison of bakers/pastry-makers and hairdressers since the physical and chemical nature of the agents involved in the job processes and possibly the underlying mechanisms of asthma development differ substantially. Moreover, for the first time, nutritional factors were assessed as potential risk factors for OA. Food Frequencies Questionnaires are considered to be reproducible and to provide a useful scale for categorizing individuals according to their intake of energy and nutrients over one year [18,43]. Although respiratory symptoms had begun up to 7 years prior to the medical visits, food habits are not likely to have changed so that the data provided by the questionnaires is a good proxy for nutrient intake at the time OA subjects became cases.

## Conclusion

This study suggests that the role of several factors that influence the incidence of occupational asthma, including dietary vitamins, might vary across exposure settings. Asthmatic cases declared higher intakes of vitamins A and D than controls, among hairdressers. The risk of OA also increases with intensity of exposure and with the body mass index in this predominantly female group. Among bakers and pastry makers, no other risk factor than the atopic status emerged.

## Competing interests

The authors declare that they have no competing interests.

## Authors’ contributions

TR and DSA carried out the epidemiologic study. They participated in the medical visits and performed the statistical analysis. CP and DZN designed the study and participated to the interpretation of results. All co-authors wrote and approved the final manuscript.

## Pre-publication history

The pre-publication history for this paper can be accessed here:

http://www.biomedcentral.com/1471-2458/12/387/prepub

## Supplementary Material

Additional file 1 **Table S1. Metric for exposure intensity: variables coding for each task in the bakery and pastry-making sectors.** Table S2. Metric for exposure intensity: variables coding for each task in the hairdressing sector. Click here for file
