# Supplementary material for: Diet, occupational exposure and early asthma incidence among bakers, pastry makers and hairdressers
Source: BMC Public Health. 2012 May 29;12:387. doi: 10.1186/1471-2458-12-387 (PMC3408355; doi:10.1186/1471-2458-12-387)
Supplement: Additional file 1 — Table S1. Metric for exposure intensity: variables coding for each task in the bakery and pastry-making sectors. Table S2. Metric for exposure intensity: variables coding for each task in the hairdressing sector. [file 1471-2458-12-387-S1.doc]

Table 1: Metric for exposure intensity*:* variables coding for each task in the bakery and pastry-making sectors
(Data were obtained from the 148 bakers and pastrymakers who performed the medical visit)

|  | Average frequency / Time spent (per day) | | |
| --- | --- | --- | --- |
| Tasks | 1^st^ Tertile (= 0) | 2^nd^ tertile 2 (= 1) | 3^th^ tertile 3 (= 2) |
| Kneading-machine loading ^†^ | <2 | ≥ 2 and < 6 | ≥ 6 |
| Transfer by shovel | < 5 | ≥ 5 and < 20 | ≥ 20 |
| Bread put in the oven ^§^ | < 7 | ≥ 7 and < 30 | ≥ 30 |
| Pastry preparation | < 5 | ≥ 5 and < 10 | ≥ 10 |
| Cleaning activities ^φ^ | < 7 | ≥ 7 and < 14 | ≥ 14 |
| Dough division | < 20 min | ≥ 20 and < 60 min | ≥ 60 min |
| Dough shaping | < 60 min | ≥ 60 and < 120 min | ≥ 120 min |

^†^ per bag, per hopper or per box

^§^ expressed in rack

^φ^ including workstation, oven, local and flour storage location cleaning

Table 2: Metric for exposure intensity*:* variables coding for each task in the hairdressing sector

(Data were obtained from the 114 hairdressers who performed the medical visit)

|  | Average frequency / Time spent (per day) | | |
| --- | --- | --- | --- |
| Tasks | 1^st^ Tertile (= 0) | 2^nd^ tertile 2 (= 1) | 3^th^ tertile 3 (= 2) |
| Perm ^†^ | <1 | ≥ 1 and < 2 | ≥ 2 |
| Hair dye ^†^ | < 6 | ≥ 6 and < 10 | ≥ 10 |
| Hair bleached ^†^ | < 4 | ≥ 4 and < 8 | ≥ 8 |
| Rinsing ^§^ | < 6 | ≥ 6 and < 10 | ≥ 10 |

^†^ sum of preparations and applications

^§^ of hair techniques (perm, hair dye and hair bleached)
